# Protective Role of Hypothermia Against Heat Stress in Differentiated and Undifferentiated Human Neural Precursor Cells: A Differential Approach for the Treatment of Traumatic Brain Injury

**DOI:** 10.29252/NIRP.BCN.8.6.453

**Published:** 2017

**Authors:** Sandeep Kumar Vishwakarma, Avinash Bardia, Nusrath Fathima, Lakkireddy Chandrakala, Syed Rahamathulla, Nagarapu Raju, Gunda Srinivas, Avinash Raj, Annamaneni Sandhya, Vishnupriya Satti, Santosh Kumar Tiwari, Syed Ameer Basha Paspala, Aleem Ahmed Khan

**Affiliations:** 1. Central Laboratory for Stem Cell Research and Translational Medicine, Centre for Liver Research and Diagnostics, Deccan College of Medical Sciences, Hyderabad, India.; 2. Department of Genetics, Faculty of Science, Osmania University, Hyderabad, India.; 3. Centre for Cellular and Molecular Biology, Hyderabad, India.

**Keywords:** Hypothermia, Neurospheres development, Neuronal phenotype, HSP-70 expression

## Abstract

**Introduction:**

The present study aimed to explore protective mechanisms of hypothermia against mild cold and heat stress on highly proliferative homogeneous human Neural Precursor Cells (NPCs) derived from Subventricular Zone (SVZ) of human fetal brain.

**Methods:**

CD133+ve enriched undifferentiated and differentiated human NPCs were exposed to heat stress at 42°C. Then, Western-blot quantification was performed using Hsp-70 (70 kilodalton heat shock proteins) recombinant protein. Finally, changes in pluripotency and Hsp-70 expression were measured using immunofluorescence staining and RT-qPCR (Quantitative reverse transcription PCR) analysis, respectively.

**Results:**

Heat stress resulted in abnormal neurospheres development. The apoptosis rate was enhanced during long-term in vitro culture of neurospheres. Neurogenic differentiation reduced and showed aberrent phenotypes during heat stress. After hypothermia treatment significant improvement in neurospheres and neuronal cell morphology was observed.

**Conclusion:**

Mild-hypothermia treatment induces attenuated heat shock response against heat stress resulting in induced HSP-70 expression that significantly improves structure and function of both undifferentiated human NPCs and differentiated neurons.

## 1. Introduction

Recently, many deaths have been reported across the globe due to hyperthermia and heat-related illnesses resulting in great medical and social hitches (Rumana, Gopinath, Uzura, Valadka, & Robertson 1998; Sharma, 2006). These stressful stimuli causes adverse effects in cells proliferation and differentiation (Morimoto, 2006). However, the detailed possible mechanisms and therapeutic measures have not been investigated. During major injury, brain is highly sensitive and vulnerable to small variations of temperature (Sharma, 2006). Recently hypothermia is gaining popularity in emergency clinics as a novel therapeutic modality for brain damage (Drury, Gunn, Bennet, & Gunn, 2014). In clinics, hypothermia has been employed in heart and brain surgery and in organ preservation to be used for transplantation (Schmitt, Tong, & Berger 2014; Li & Yang, 2014). Very little is explored about adaptive thermogenesis against the heat and cold shock response in mammalian brain cells. And the search is still on to identify the neurotoxic effect of temperature related stress on brain cells.

Earlier studies have demonstrated activation of stress response and apoptotic cell death during temperature mediated stress in various types of cells (Watanabe & Okada, 1967; Sharp & Sagar 1994; Vania & Ian, 2002; Sharma & Hoopes, 2003; Yao et al., 2011). Changes in cellular milieu due to temperature stress in brain may include the free radical generation, altered efflux mechanisms, abnormal or depressed neuronal protein synthesis, and alterated gene expression. The time course and gene expression profile may vary depending upon the nature of insult and type of cells involved. So far, the role of temperature induced mechanisms has not been elucidated in homogenous population of human Neural Precursor Cells (NPCs) during long-term exposure. Hence, it is of utmost importance to explore the basic cellular and molecular mechanisms underlying the harmful and beneficiary effects of hyperthermia and hypothermia on human NPCs population and its lineages. In addition, monitoring the cellular and molecular changes may provide a powerful tool to understand the mechanisms involved in stress response in neuronal cell type.

Previous studies have reoprted the defense mechanisms during the deliterious consequences of interactions and abnormal proteins folding in brain cells. Heat shock proteins-70 (Hsp-70) are well known chaperon molecules which asssist proper folding and transportation of various proteins (Morimoto, Tissieres, & Georgopoulos 1994; Welch & Gambetti, 1998; Yenari, Giffard, Sapolsky, & Steinberg 1999; Mosser et al., 2000; Westerheide & Morimoto 2005). However their expression patterns against heat and mild cold stress response in human NPCs and its lineages has not been identified yet. Thus, identifying the expression of such molecules and their correlation with pluripotent markers of human NPCs will provide a new insight to better understand the effect of temperature stress on their regenerative potential.

NPCs have already proved their potential to serve as the vehicle for replenishment and repair of Central Nervous System (CNS) tissues (Paspala, Vishwakarma, Murthy, Rao, Khan, 2012; Vishwakarma et al., 2013; Vishwakarma, Paspala, Tiwari, & Khan; 2014). However changes in body temperature might be associated with certain neurodegenerative conditions due to death of residing cells in the brain tissues and in turn, resulting in tissue damage (Hochachka, 1986; Fijita, 1999; Mrozek, Vardon, & Geeraerts 2012). Such adverse condition requires assisstence of stem cells to repair the damage.

Logically, to fulfill this task endogenous NPCs residing in human brain should not be damaged due to unfavourable conditions (such as higher and lower temperatures). Hence, we hypothesized that human NPCs must be heat and cold tolerant during long-term in vitro exposure. To test this hypothesis, we enriched NPCs derived from Subventricular Zone (SVZ) of human fetal brain using prominin-1 (CD133) cell surface antibody. The heat and cold tolerance of CD133+ve enriched cells was determined by comparing their in vitro proliferation and differentiation potential at heat (42°C) and mild cold shock (33°C) treatment versus the normal growth temperature (37°C) at different time points. After the treatments, the resultant cells were assessed for their pluripotency, stress response, growth pattern, and changes in gene transcripts expression.

## 2. Methods

### 2.1. Isolation and enrichment of hNPCs derived from SVZ of human fetal brain

Third trimester (12 gestation weeks, N=2) human fetal SVZ tissues (spontaneously aborted) were dissected out from the brain and dissociated under sterilized conditions using mechanical and enzymatic digestion method as described previously (Vishwakarma et al., 2013). Single cell suspension was prepared after filtering through 40 μm cell strainer. Trypan Blue Exclusion assay was used to determine the percentage of cell viability. The discrimination between percentage of viable and dead cells was made by cell counting using hemocytometer. Enrichment of human NPCs was performed by Magnetic Activated Cell Sorting (MACS) using CD133 antibody as per the manufacturer’s instructions (Milteny Biotec, Germany). CD133+ve enriched cells were used for further experimental purposes.

### 2.2. In vitro proliferation and differentiation

CD133+ve enriched precursor cells were cultured as suspension in human neural proliferation medium (Stem Cell Technologies, Canada) supplemented with basic-Fibroblast Growth Factor (b-FGF, 10 ng/mL), Epidermal Growth Factor (EGF, 20 ng/mL) and 1X antibiotic and antimycotic solution. Cells in proliferation medium were incubated at 37°C with 5% CO_2_ atmosphere for 14 days following 50% medium change every after the third day of culture. Development of neurospheres was observed and dissociated at day 14 followed by single cell suspension. Next, 4 X 103 cells derived from developing primary neurospheres at day 14 were cultured on 0.2% gelatin coated cover slips in human neuronal differentiation medium (Stem Cell Technologies, Canada) supplemented with 2% Fetal Calf Serum (FCS) and retinoic acid. Mitogenic growth factors were withdrawn during differentiation. Cells were allowed to differentiate for 14 days at 37°C and 5% CO_2_. Fresh complete neural differentiation medium was replenished every after the third day of initial seeding.

### 2.3. Cells proliferation and differentiation under heat stress

CD133+ve enriched human NPCs during in vitro proliferation were exposed to heat stress at 42°C for 14 days. Control cells were cultured at 37°C and assessed for their potential to generate secondary and tertiary neurospheres and further compared with the treated cells at day 14. The primary neurospheres developed in control condition (without heat/or cold stress) were dissociated at day 14 of culture and further cultured in human neural differentiation medium without mitogenic factors as described above. These cultures were allowed to differentiate for 14 days for the assessment of alterations at cellular and molecular levels.

### 2.4. Mild hypothermia treatment of cells under heat stress

To identify the effect of mild hypothermia (33°C) on human neurological cells both undifferentiated and differentiated cells were exposed to heat shock at 42°C for 2 h and then subjected to mild hypothermia at 33°C for 6 h.

### 2.5. Detection of heat inducible Hsp-70 and constitutive Hsc-70 protein expression by Western blot analysis

Western-blot quantification was performed in both undifferentiated and differentiated human neurological cells using Hsp-70 recombinant protein that recognizes both Hsp-70 and Hsc-70 proteins. A rabbit polyclonal antibody (SPA816, Strassgen) was used to identify 73 kDa Hsc-70 protein. Membrane was incubated overnight with primary antibody followed by two hours incubation with secondary antibody at room temperature. The immunoblot was probed using Millipore Immobilon Western blot reagent.

### 2.6. Reactive oxygen species estimation

Heat stressed cells during in vitro culture along with controls were harvested at day 14 from the culture plates. Undifferentiated and differentiated cells during 2 h of heat stress and 6 h of post-hypothermia were also used for (Reactive oxygen species) ROS estimation to determine the alterations due to hypothermia treatment. Single cell suspension was prepared and further stained with 5(6)-Carboxy-2′,7′-Dichlorofluorescein (DCFDA) for ROS estimation. Intensity of fluorescence was measured by Cell Quest software in flow cytometry (Becton Dickinson).

### 2.7. Immunofluorescence staining

Immunofluorescence staining was performed by fixing cells in 4% Paraformaldehyde (PFA) for 30 min at 4°C. For intracellular staining, cells were permeabilized for 15 min at room temperature using Triton-X-100 (0.1%). Following the permeabilization, cells were washed twice using 1X PBS. Undifferentiated cells were stained with anti-human Nestin-PE (1:200, R&D System), whereas differentiated cells were stained with β-tubulin-III-PE (1:200) overnight at 4°C in dark. Post-staining cells were washed twice with 1X PBS to remove the unbound antibodies. Cells were mounted and imaged using fluorescence microscope (Carl Zeiss, Germany).

### 2.8. Morphometric assessment post-hypothermia

The morphometric analysis of developing neuro-spheres and differentiated neurons was performed by phase contrast microscopy pre- and post-hypothermia. Total number of secondary and tertiary neurospheres was determined by counting neurospheres per field. Changes in neuronal morphology, dendritic length and number of dendrites per cell were identified and compared with control and heat stressed cells.

### 2.9. Assessment of pluripotent markers and stress proteins in proliferating and differentiating cells using RT-qPCR

Ribonucleic Acid (RNA) was isolated from the cells in each group using standard Guanidium Isothiocyanate (GITC) protocol with minor modifications. RNA was quantified and complementary DNA (cDNA) was prepared using reverse-transcriptase II (Invitrogen). About 2 μL of cDNA was used for measuring the relative quantification of mRNA transcripts using SYBR-Green based RT-q-PCR. Primers specific for neural multipotent markers (Nestin), major ABC transporters (ABCB1 and ABCG2) and Hsp-70 (HSPA4, HSPA9 and HSPA14) were used to quantify their expression pattern during stress and hypothermia treatment. GAPDH gene specific primers were used for normalization of test samples. Ct values of all the transcripts were noted and used for statistical analysis for fold difference calculation. All reactions were performed in triplicates to avoid any technical error.

### 2.10. Statistical analysis

Graph Pad Prism software (version V) was used for statistical analyses, including ANOVA. Multiple comparison was used to analyze one-way and two-way variance and was corrected by the Bonferroni post hoc test. Unpaired t test was used to compare mean values. Microsoft Office Excel (2007) was used for statistical computing and graphical representation of variance. RT–qPCR efficiency was determined by StepOne software (version 2.2). The regression value (R^2^) of ≥0.99 was considered significant with 100% PCR efficiency. Experimental significant level was determined by P≤0.05 for all the variables.

## 3. Results

### 3.1. Imunomagnetic sorting and in vitro culture establishemnt

**Figure 1.**
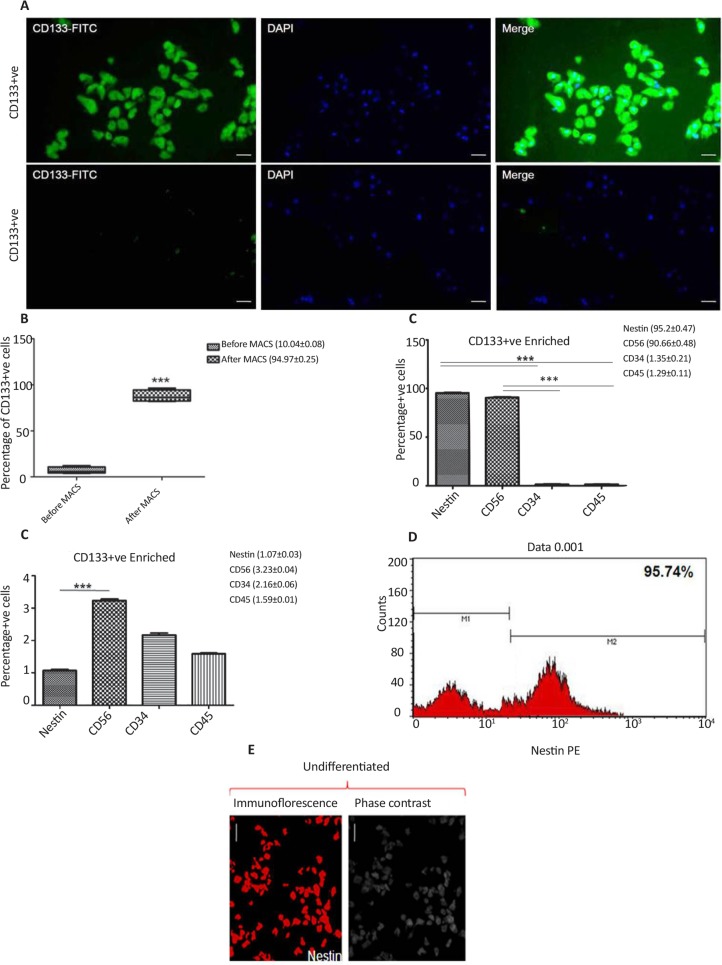
(A) Immunofluorescence staining showing green fluorescence for CD133+ve enriched cells. DAPI was used as counter stain to stain the cell nuclei; (B) Bar graphs showing percentage of CD133+ve cells before and after MACS; (C) Percentage of neural cell specific markers (Nestin and CD56) and negative markers (CD34 and CD45) in CD133+ve and CD13-ve enriched cell population; (D) Flow cytometry histogram showing percentage of cells positive for nestin in CD133+ve enriched cell population; (E) Phase contrast and immunofluorescence staining of CD133+ve enriched undifferentiated neural cells with nestin

**Figure 2.**
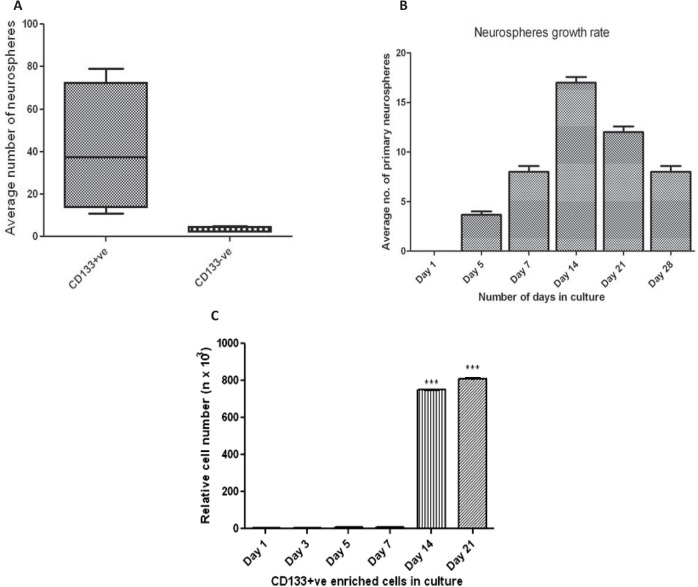
(A) Average number of neurospheres formed by CD133+ve and CD133-ve enriched cells; (B) Neurospheres growth rate during in vitro development of CD133+ve enriched cells derived primary neurospheres from day 1 to day 28; (C) Increase in relative cell number during in vitro expansion of CD133+ve enriched cells

Immunomagnetically sorted cells isolated from SVZ of human fetal brain showed significantly higher staining for CD133 antibodies (Figure 1A). More than 90% of cells were found positive for CD133 post-MACS (Figure 1B). The immunophenotypic analysis of pluripotent markers in CD133+ve and CD133-ve enriched cells population revealed significanlty higher percentage of cells expressing neural specific markers (nestin and CD56) in CD133+ve enriched cells as comapred to the CD133-ve populaiton (Figure 1C). The percentage cells expressing hematopoeitic lineage markers were quite less in both the enriched cells popualtion. The existence of neural precursor cells in CD133+ve enriched cells popualtion was further verified by ICC staining (Figure 1D & 1E). In vitro neurospheres development ability of CD133+ve was significantly higher than the CD133-ve enrcihed cells (Figure 2A). Hence, the further analysis was performed for CD133+ve enriched cells. The growth rate of developing neurospheres derived from CD133+ve cells was the highest at day 14 of culture (Figure 2B). The relative viability of cells within the developing neuro-spheres was highest at day 14 and 21 (Figure 2C).

### 3.2. Recovery in developing neurospheres and neuronal differentiation post-hypothermia

**Figure 3.**
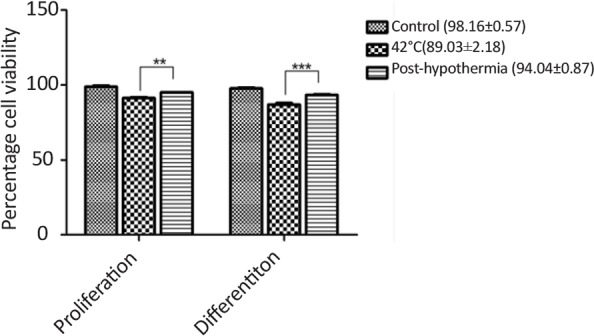
Changes in percentage cell viability post-hypothermia followed by hyperthermia treatment

**Figure 4.**
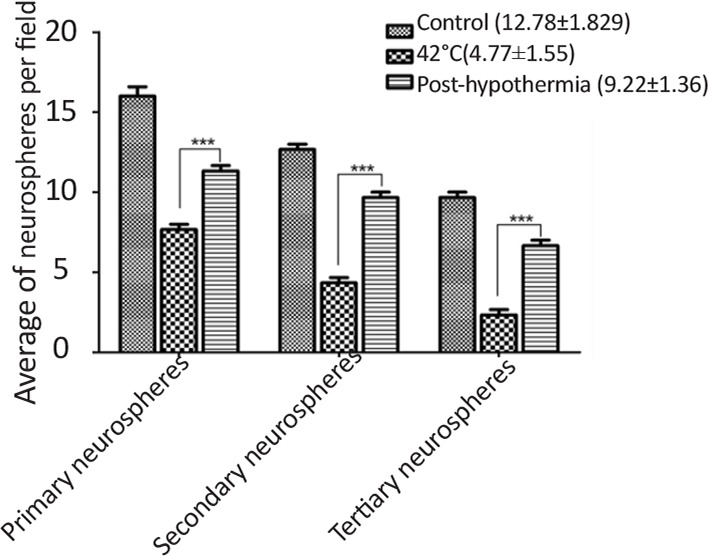
Changes in average number of neurospheres formed post-hypothermia followed by hyperthermia treatment

Heat stress resulted in reduced cell viability during proliferation as well as differentiation of CD133+ve enriched cells which signifcantly incerased after hypothermia treatment (Figure 3). Heat stress also resulted in increased number of primary neurospheres development, however, reduced number of secondary (2°) and tertiary (3°) neurospheres were generated during sub-culturing as compared to control (Figure 4). The irregular larger and uneven size of neurosperes were generated during heat stress. The neurogenic lineage differentiation also reduced and displayed aberrent phenotype.

**Figure 5.**
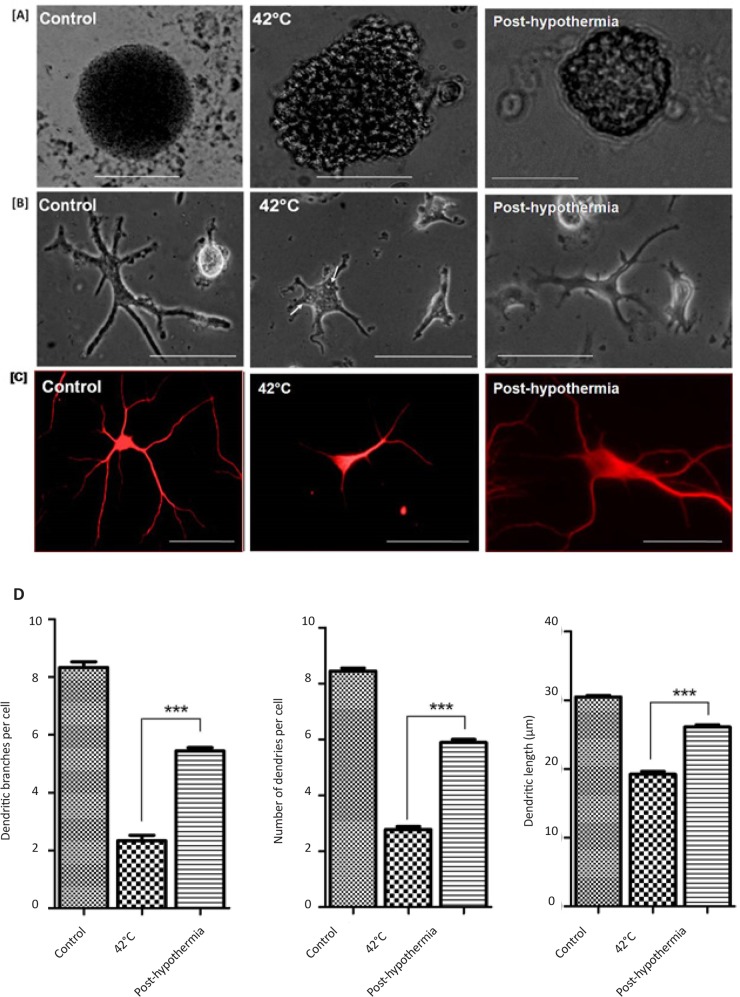
Changes in cellular phenotype in developing (A) neurospheres and (B) differentiating neurons under heat stress followed by hypothermia treatment. (C) Immunofluorescence staining of neurons with β-tubulin III antibody to characterize the changes in neuronal cell phenotype in three different conditions. Significantly improved number and phenotypes of neurons was observed post-hypothermia treatment as compared to heat stressed cells (*P<0.01; scale bar=100 μm).

Neurons generated under stress condition were smaller in size as compared to control. The neurons had fewer number of dendritic branched and shorter dendritic length. Neuronal differentiation was slow during heat stress as compared to controls. Large number of stress grannules were also observed in developing neurons (indicated by arrow) during heat stress. These phenotypic alterations were reverted to almost normal after hypothermia treatment (Figure 5). Significant post-hypothermia treatment improvement of neurospheres morphology was observed after 14 days of proliferation (Figure 5A). As such hypothermia also resulted in improved morphology of developing neurons during differentiation (Figure 5B & 5C). The number and size of neuronal dendrites was also improved post-hypothermia (Figure 5D).

### 3.3. Attenuated heat shock response and hypothermia

**Figure 6.**
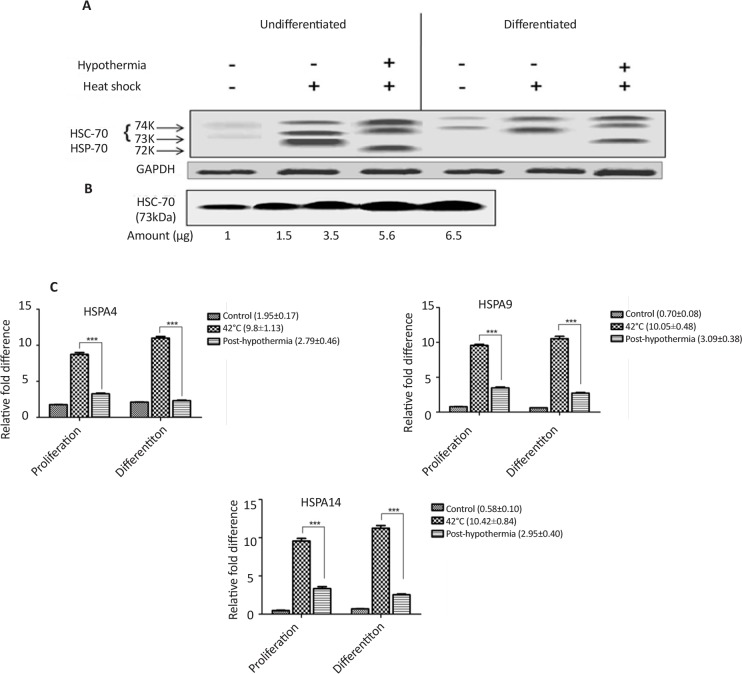
Attenuated induction in Hsp-70 protein synthesis and enhanced constitutive Hsc-70 protein expression in undifferentiated and differentiated cells (A) Western blot results for Hsp-70 and Hsc-70 during proliferation and differentiation in response to heat shock at 42°C for 3 h and recovered cells following mild hypothermia (33°C) treatment for 6 h; (B) Immunoblotting of Hsc-70 protein expression to identify the fold expression of Hsc-70 in control, heat shocked and post-hypothermia treated cells; (C) RT-qPCR analysis for mRNA expression of Hsp-70 transcripts (Hspa-4, Hspa-9 and Hspa-14) during heat and post-mild hypothermia treatment in undifferentiated and differentiated cells

Hsp-70 protein expression was analyzed in developing neurospheres and lineage differentiation under heat stress to investigate the cellular and molecular mechanisms in human neurological cells. Western blot results showed decreased induction of Hsp-70 (72 kDa) expression in neuronal cells as compared to neurospheres derived cells (Figure 6A & 6B). This decreased induction in Hsp-70 expression was further validated by mRNA expression analysis using RT-qPCR at 42°C of heat stress and after recovery at mild cold temperature (33°C). Figure 6C clearly shows the induced expression of HSP-70 mRNA transcripts after hypothermia treatment.

### 3.4. Nestin and β-tubulin-III expression enhance post-hypothermia

**Figure 7.**
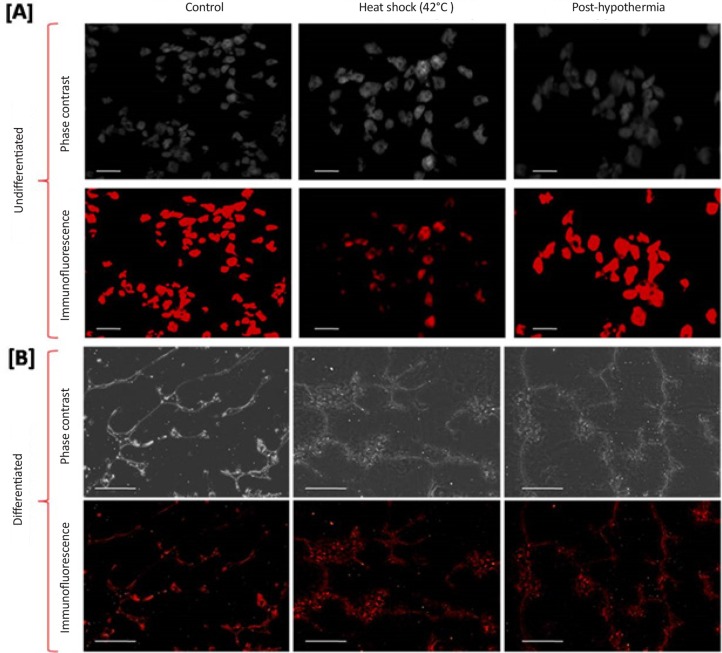
ICC staining of differentiated cells in response to heat stress showing the changes in expression of nestin in control, heat shocked and hypothermia treated cells during proliferation (scale bar=50 μm) and β-tubulin-III during neuronal differentiation (scale bar=100 μm)

Induction in Hsp-70 in particular has demonstrated to confer the cytoprotection. Whereas vulnerability to attenuated Heat Shock Response (HSR) in pluripotency of undifferentiated and differentiated cells against forced Hsp-70 expression needs to be rectified. Hence we examined nestin expression in undifferentiated cells before and after heat stress following hypothermia treatment. Reduced expression was observed for nestin expression during heat shock and was later recovered to some extent after hypothermia (Figure 7A). Immunofluorescence micrographs of differentiated cells showed eminent expression for neuronal specific marker β-tubulin-III after 14 days post-recovery (Figure 7B).

### 3.5. Changes in redox balance and cellular apoptosis post-hypothermia

**Figure 8.**
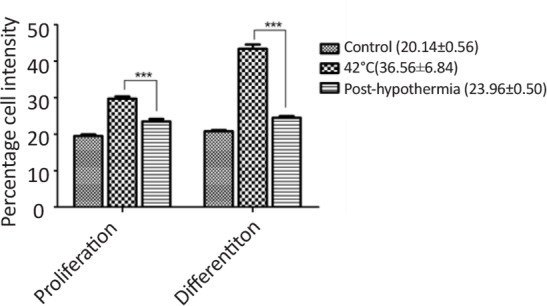
Flow cytometry analysis showing variations in ROS generation post-hypothermia in undifferentiated and differentiated cells

**Figure 9.**
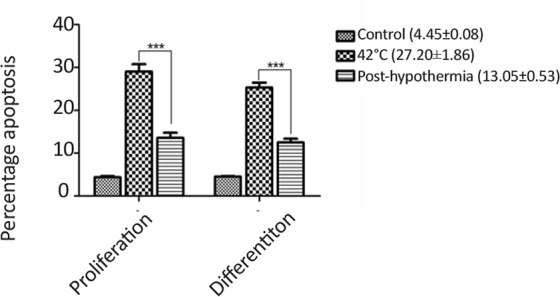
Bar graphs representing the changes in percentage cell apoptosis post-hypothermia following to heat stress

More often, neurogenic lineage differentiation is associated with the enhanced oxidative stress, which plays an important role to integrate the neurospheres development and lineage differentiation. For this reason, we estimated the redox balance post-hypothermia in proliferating and differentiating cells exposed to heat stress. Figure 8 shows decreased redox imbalance post-hypothermia in differentiated neurogenic cells when compared to the undifferentiated neural cells. The cellular apoptosis level was also reduced post-hypothermia during proliferation as well as differentiation (Figure 9).

### 3.6. Molecular profiling

**Figure 10.**
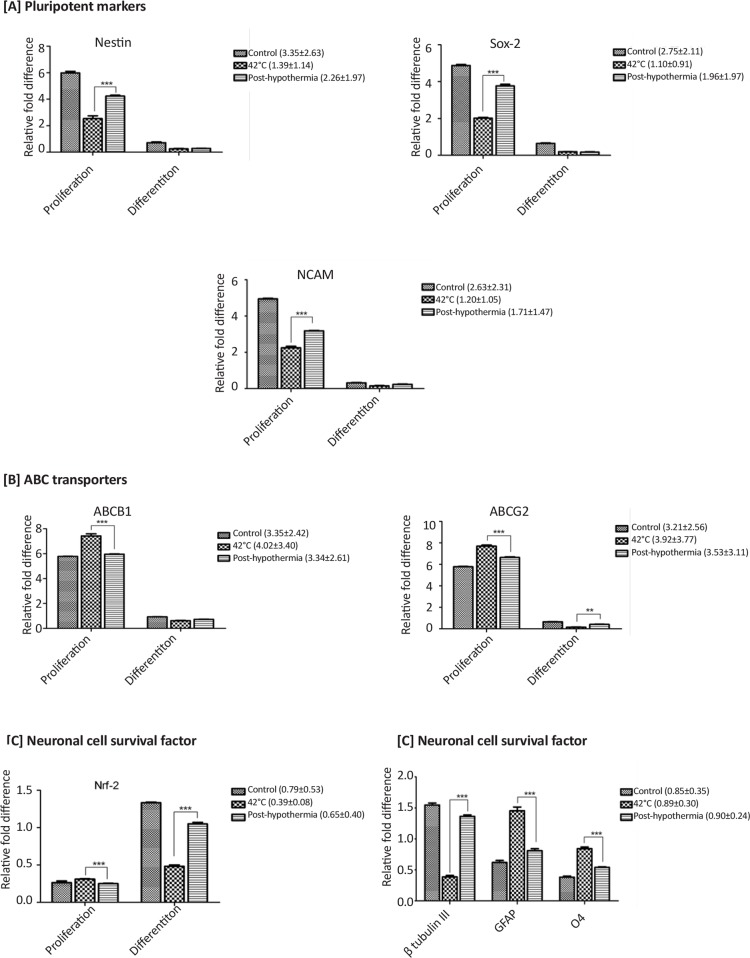
RT-qPCR analysis showing the changes in fold expression of respective gene transcripts of (A) neural precursor markers (Nestin, Sox-2 and NCAM), (B) drug transporters (ABCB1 and ABCG2), and (C) cell survival factor (Nrf-2) during proliferation and differentiation. (D) Molecular changes in neural lineage specific markers (β-tubulin-III, GFAP and O4) during differentiation of neural precursor cells post-hypothermia followed by exposure to heat stress

Gene expression analysis of mRNA transcripts for pluripotent markers (Nestin, Sox-2 and NCAM) showed reduced expression during stress and enhanced post-hypothermia treatment (Figure 10A). ABC transporters (ABCB1 and ABCG2) showed increased expression during heat stress and got diminished post-hypothermia in both conditions (i.e. proliferation and differentiation) (Figure 10B). Continous increase in Nrf-2 expression during proliferation on exposure to heat stress showed commitment of cells towards maturation (Figure 10C). However, during differentiation decreased expression of Nrf-2 mRNA transcripts during stress condition reflects defects in neuronal cell phenotype. Complete absence of neural lineage markers (β-tubulin III for neurons, GFAP for astrocytes and O4 for oligodendrocytes) expression was observed during proliferation of CD133+ve enriched cells. During differentiation decreased level of mRNA transcripts of neural lineage marker (β-tubulin III) was observed on exposure to heat stress. However increased glial differentiation (GFAP and O4 expression) was observed during stress. Hypothermia treatment resulted in significant improvement in the expression of β-tubulin III whereas the expression levesl of glial markers decreased (Figure 10D).

## 4. Discussion

This study explores some of the crucial mechanisms involved in cellular and molecular deficits during heat and mild cold stress on undifferentiated and differentiated human NPCs derived from SVZ of human fetal brain during long-term in vitro culture. Since multipotent brain cells (NPCs) in different niches are responsible for correcting damages against such stressful stimuli, studying the direct effect of cellular and molecular consequences in homogenous population of undifferentiated and differentiated NPCs will provide better insight for the development of new therapeutic strategies to overcome on temperature mediated stress response. However, these is no appropriate model available to identify such changes in human NPCs and its lineages providing closer imminent of cellular deficites within the human brain. To our knowledge, this is the first study exploring the major cellular and molecular alterations happening in homogenous primary human NPCs population.

As per enhanced cell cycle activity during heat stress, the shape of developing neurospheres was altered slightly and increased in size representing cells shifting towards the maturation and differentiation. However, induced glial differentiation was observed during stress conditions representing abnormal differentiation with distorted cell phenotype. Slow neuronal growth was observed due to stress with swollen and condensed phenotype. Presence of stress grannules in neurons is an indication of stress-induced cell response. Increased ROS level on exposure to heat stress reflected defects in protein folding due to stress conditions. Higher percentages of cell intensity during differentiation than proliferation represents enhanced stress responsible for more phenotypic damage.

ABC transporters are membrane proteins involved in stress resistance against various types of drugs/stimuli. Some transporters are expressed more during stem cells proliferation whereas downregulation of such transporters idicates cellular maturation and differentiation. Over-expression of ABCB1 and ABCG2 in undifferentiated NPCs during heat stress represents resistance against stress stimuli. Irrespective to this downregulation in pluripotent marker (Nestin) against stress shows degeneration in pluripotency of NPCs.

Hsp-70 is a stress protein, which plays major role in modulating stem cell behavior against stressful conditions (Sherman & Goldberg, 2001; Fan, 2012). This was further proved by over expression of Hsp-70 mRNA transcripts during stress suggesting their defense mechanisms against damage or improper protein foldings. Thus, Hsp-70 might be considered as one of the major players in defense against stress and other harmful conditions to the cells. Hence, regulation of Hsp-70 expression in undifferentiated and differentiated human neurological cells was established by using mild hypothermia (33°C) treatment of cells after exposure to heat stress at 42°C.

We observed that both differentiated and undifferentiated cells are associated with an attenuated heat shock response. Figure 6 clearly shows decreased induction in Hsp-70 protein expression levels in differentiated human neuronal cells when compared with the neurospheres derived undifferentiated precursor cells. These results are consistent with previos studies of reduced Hsp-70 induction during differentiation in different neuronal cell lines (Nelson, Kruuv, Koch, & Frey 1971; Hatayama, Tsujioka, Wakatsuki, Kitamura, & Imahara 1992; Liu, Bian, Huang, & Lee, 1994; Hatayama, Takahashi, & Yamagishi, 1997; Oza, Yang, Chen, & Liu 2005). These studies in different neuronal system demonstrated variable heat stress response providing an evidence for defect in attenuated activation of heat shock factor-1 (Tanaka et al., 2009).

Our study revealed that protective Heat Shock Resposnse (HSR) is limited to differentiating neurons which results in altered neuronal cell phenotype. This limited ability of neuronal cells to mount protective HSR have fundamental role in the pathogenesis of various neuro-degenerative conditions (Gonzalez et al., 1989; Kinouachi et al., 1993; Rajdev & Sharp, 2000). However, the exact mechanism responsible for the attenuated HSR in undifferentiated and differentiated neurological cells is not clearly understood. Hence, to betetr understand the cellular and molecualr mechanisms involved in altering the expression levels of Hsp-70 transcripts, we analyzed Hsp-70 protein expression under stress conditions and compared with the normal conditions.

We found reduced level of Hsp-70 expression of proteins in both conditions of post-hypothermia teratment against heat stress (Figure 6). In addition to this, expression of Hsc-70 cannot be ignored. Hsc-70 has been found to guide sequential restructuring of transient or stable protein complexes by interacting with various Co-Chaperon proteins. This promotes temporal and spatial regulation of exo- and endo-cytotic machinary of protein transport and regulated assembly and disassembly of protein complexes. However in this study, reduced expression of Hsc-70 during neuronal differentiation demonstrates recovery from heat stress due to hypothermia treatment. These findings are well supported by previous published reports (Soloff, Nagle, Moss, Henle, & Crawford 1987; Hatayama, Takahashi, & Yamagishi, 1997; Oza et al., 2005; Polderman, 2008).

Induction in Hsp-70, in particular, has demonstrated to confer the cytoprotection in many studies (Gabai & Sherman, 2002; Chen & Brown, 2007; Zeng, Kulkarni, Inoue, & Getzenberg, 2009). Whereas vulnerability to attenuated HSR in pluripotency of undifferentiated cells against forced Hsp-70 expression needs to be rectified. Hence we examined expression of pluripotent marker, nestin, in undifferentiated cells before and after heat stress following hypothermia treatment. Induced expression of nestin in NPCs post-hypothermia demonstrated significant improvements in pluripotency during heat stress (Figure 10A), which was also supported by the development of secondary and tertiary neurospheres. This is another evidence for recovery in developing neural cell phenotypes. As such hypothermia also resulted in improved morphology of developing neurons with improved number and size of dendrites during differentiation (Figure 5).

Further β-tubulin III expression proved improvement in differentiating neurons under heat stress following hypothermia treatment (Figure 10D). Decreased ROS production and reduced neuronal cell death provides significant evidence of mild hypothermia treatment as an effective protector of brain cells injury/stress. Few studies have demonstrated vascular cooling as the most effective strategy to reduce infarct volume and functional outcome (Van Rijn, Van den Berg, Kipp, Schamhart, & Van Wijk, 1985; Wang et al., 2010). This study well supports our findings in all aspects of structural and molecular deficits produced by heat stress in immature NPCs and differentiated neuronal cells.

In summary, our study provides evidence that changes in the expression of Hsp-70 is an important aspect during heat and mild-cold stress. Mild hypothermia treatment induces attenuated HSR against heat stress resulting in reduced induction in Hsp-70 expression by activating regenerative mechanisms that significantly improves structure and function of both undifferentiated human NPCs and differentiated neurons. However, more studies are required to determine the exact targets, time duration for hypothermia treatment in different conditions to confirm its significant role as consistent protective agent against cerebral ischemia and other neurological deficits.
